# Neural Plastic Changes in the Subcortical Auditory Neural Pathway after Single-Sided Deafness in Adult Mice: A MEMRI Study

**DOI:** 10.1155/2018/8624745

**Published:** 2018-11-26

**Authors:** So Young Kim, Hwon Heo, Doo Hee Kim, Hyun Jin Kim, Seung-ha Oh

**Affiliations:** ^1^Department of Otorhinolaryngology-Head & Neck Surgery, CHA Bundang Medical Center, CHA University, Seongnam, Republic of Korea; ^2^Department of Radiology, Seoul National University Hospital, Seoul, Republic of Korea; ^3^Department of Otorhinolaryngology-Head & Neck Surgery, Seoul National University Hospital, Seoul, Republic of Korea; ^4^Department of Brain Science, Seoul National University, Seoul, Republic of Korea

## Abstract

Single-sided deafness (SSD) induces cortical neural plastic changes according to duration of deafness. However, it is still unclear how the auditory cortical changes accompany the subcortical neural changes. The present study aimed to find the neural plastic changes in the cortical and subcortical auditory system following adult-onset single-sided deafness (SSD) using Mn-enhanced magnetic resonance imaging (MEMRI). B57BL/6 mice (postnatal 8-week-old) were divided into three groups: the SSD-4-week group (postnatal 12-week-old, n = 11), the SSD-8-week group (postnatal 16-week-old, n = 11), and a normal-hearing control group (postnatal 8-week-old, n = 9). The left cochlea was ablated in the SSD groups. White Gaussian noise was delivered for 24 h before MEMRI acquisition. T_1_-weighted MRI data were analyzed from the cochlear nucleus (CN), superior olivary complex (SOC), lateral lemniscus (LL), inferior colliculus (IC), medial geniculate body (MG), and auditory cortex (AC). The differences in relative Mn^2+^-enhanced signal intensities (Mn^2+^SI) and laterality were analyzed between the groups. Four weeks after the SSD procedure, the ipsilateral side of the SSD showed significantly lower Mn^2+^SI in the CN than the control group. On the other hand, the contralateral side of the SSD demonstrated significantly lower Mn^2+^SI in the SOC, LL, and IC. These decreased Mn^2+^SI values were partially recovered at 8 weeks after the SSD procedure. The interaural Mn^2+^SI differences representing the interaural dominance were highest in CN and then became less prominently higher in the auditory neural system. The SSD-8-week group still showed interaural differences in the CN, LL, and IC. In contrast, the MG and AC did not show any significant intergroup or interaural differences in Mn^2+^SI. In conclusion, subcortical auditory neural activities were decreased after SSD, and the interaural differences were diluted in the higher auditory nervous system. These findings were attenuated with time. Subcortical auditory neural changes after SSD may contribute to the change in tinnitus severity and the outcomes of cochlear implantation in SSD patients.

## 1. Introduction

Recently, the treatment of single-sided deafness (SSD) has extended to cochlear implants (CIs), in addition to contralateral routing of signal (CROS) or a bone-anchored hearing aid (BAHA). SSD patients are expected to regain binaural hearing after CI, which cannot be achieved with CROS or BAHA. An increasing number of studies reported the favorable outcomes of CI in SSD patients in aspects of speech intelligibility, sound localization, and quality of life [1, 2]. Tinnitus was also alleviated after CI in SSD [3]. However, the outcomes of CI showed differences between postlingual SSD and prelingual SSD patients [1, 4]. The differences in the experience of hearing between prelingual and postlingual SSD patients might result in different changes to the brain auditory pathways following single-sided hearing deprivation. We have demonstrated the disappearance of contralateral dominance in the auditory cortex after SSD in adult rats [5]. However, it is still elusive whether these changes in auditory cortical activities occur independently or accompany subcortical auditory neural changes.

There is limited knowledge on the changes to the central auditory pathways. Several studies have reported changes in the cortical and subcortical auditory system after SSD [6–8]. The cortical gray matter volume is reduced, and brain activities are decreased in multiple cortical areas, including the temporal gyrus, in unilateral hearing loss patients [6]. The subcortical auditory region of inferior colliculus (IC) shows decreased activities after SSD in gerbils [7]. Another study reported decreased protein synthesis after unilateral conductive hearing loss in the medial superior olive [8]. Although a number of previous studies showed cortical or subcortical auditory neural changes after SSD, no study has explored both cortical and subcortical neural plastic activity and its changes following SSD according to duration of deafness. A complex analysis that includes comparison of activation in several nuclei along the auditory pathways is necessary to understand the plastic transformations that occur as adaptations to changed asymmetric inputs.

Because the auditory nervous system has early and multiple decussations from the level of the cochlear nucleus (CN), both ipsilateral (deaf side) and contralateral (normal-hearing side) auditory neural pathways of deafness could be influenced by SSD [9]. There are effects of the ascending auditory fibers that cross over to the contralateral side at multiple levels of the auditory nervous system accumulating to the higher orders of the auditory nervous system [10]. Hence, unilateral auditory deprivation may be attributed to the functional changes along the nuclei within the auditory pathways, in accordance with decussating fibers. To explore auditory nervous decussation, SSD models should be used. Several prior studies used conductive hearing loss models, but these models could be contaminated with ipsilateral cochlear stimulation by crossed sounds [7, 8]. After SSD, the contralateral dominance of the auditory midbrain and cortex response disappears [5, 11, 12]. However, a few previous studies showed changes in subcortical auditory neural activities after cochlear ablation. Cochlear ablation can result in reduced c-fos expression in the auditory brainstem of mature animals [13]. A unilateral cochlear ablation and follow-up study of subcortical and cortical activity may help us understand the decussation pattern and its plasticity in the auditory system.

Mn-enhanced magnetic resonance imaging (MEMRI) is a promising neuroimaging tool for mapping the neural activity patterns of small animals with high resolution [14, 15]. In accordance with the uptake of Mn^2+^ ions within activated neurons via voltage-gated Ca^2+^ channels, sound-evoked neural activities can be traced retrospectively. MEMRI is superior to other neuroimaging technologies, including functional MRI, due to the higher resolution, and it can be adopted to small animals, including mice [16]. In addition, MEMRI enables visualization of deep brain lesions, such as in the IC and hypothalamus, while other optical imaging techniques, such as voltage- or calcium-sensitive dyes and tow-photon images, have limited depths of penetration [17, 18].

The present study postulated that postlingual SSD would induce subcortical as well as cortical auditory neural plastic changes. To explore this hypothesis, the auditory neural activity changes in the adult SSD mouse model were examined using MEMRI. The sound-evoked MEMRI changes were reported in the brainstem cochlear nuclei (CN) and IC [19, 20]. Only one study has investigated cortical and subcortical auditory neural activities in an adult SSD mouse model [21]. However, no study has evaluated the longitudinal changes to MEMRI after SSD, to our knowledge. In this study, sound activity-dependent Mn^2+^ uptake was measured throughout the auditory nervous system of the CN, superior olivary complex (SOC), lateral lemniscus (LL), IC, medial geniculate body (MG), and auditory cortex (AC). The relative Mn^2+^-enhanced signal intensity (Mn^2+^SI) changes were compared between the control group and the SSD (4-week and 8-week) groups. The aural dominance was examined by comparing Mn^2+^SI between ipsilateral and contralateral ears. The auditory neural plastic changes were explored by comparing the Mn^2+^SI between 4 and 8 weeks after SSD.

## 2. Methods

### 2.1. Animal Experiments

**Figure 1:**
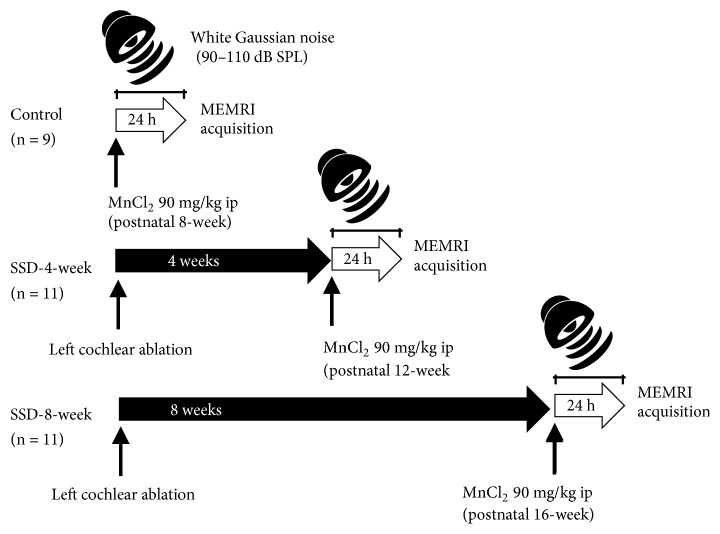
Schematic diagram of the present study. B57BL/6 mice (postnatal 8-week) were divided into three groups: (1) normal-hearing control group (n = 9, postnatal 8-week), (2) the single-sided deafness- (SSD-) 4-week group (n = 11, postnatal 12-week), and (3) the SSD-8-week group (n = 11, postnatal 16-week). After intraperitoneal injection of 90 mg/kg MnCl_2_ solution, all mice were exposed to white Gaussian noise for 24 hours. Then, MRI was acquired (ip: intraperitoneal injection).

Male B57BL/6 mice (postnatal 8-week-old) were used in the present study. All anesthetic, surgical, and postsurgical procedures used in this study, as well as animal care, were approved by the Institutional Animal Care Committee of Seoul National University of Korea (IACUC No. 15-0204-C2A4). The mice were divided into three groups: control, SSD-4-week, and SSD-8-week. The control group (postnatal 8-week-old, n = 9) mice were subjected to MEMRI immediately. The SSD-4-week group underwent left-side cochlear ablation surgery and recovered for 4 more weeks until MEMRI study (postnatal 12-week-old, n = 11). The SSD-8-week group recovered for 8 weeks after left cochlear ablation surgery before MEMRI study (postnatal 16-week-old, n = 11) (Figure 1).

The mice were anesthetized with an intraperitoneal injection of a mixture of Zoletil (30 mg/kg) and xylazine (5 mg/kg). All cochlear ablation surgeries were conducted unilaterally on the left ear. After the fur was shaved behind the left ear, a postauricular incision was made. The otic bulla was dissected with care to preserve the facial nerve. A small opening was made in the otic bulla, and the cochlea was visualized. The cochlea was punctured with a 26-gauge needle and was irrigated with kanamycin through the perforation three times. Then, the opening in the cochlea was closed with glue, and a subcutaneous 4.0 Vicryl suture was added. Hearing levels were confirmed before MEMRI acquisitions in all mice groups using the auditory brainstem response (ABR) (SmartEP, Intelligent Hearing Systems, Miami, FL, USA) as described previously [5]. The ABR results are presented in Table 1. The average ABR thresholds of the deaf side were 81.36 (standard deviation (SD) = 10.02) dB SPL and 80.00 (SD = 10.00) dB SPL for SSD-4-week and SSD-8-week groups, respectively.

### 2.2. Manganese Injection and Sound Stimuli

Control (8-week-old), SSD-4-week (postnatal 12-week-old), and SSD-8-week (postnatal 16-week-old) mice received an intraperitoneal injection of a 90 mg/kg MnCl_2_ solution diluted with normal saline. The saline (2 ml) was injected subcutaneously to maintain hydration.

All mice were placed in a cage within a soundproof box and exposed to white Gaussian noise for 24 h prior to MEMRI acquisition (Figure 1). The white Gaussian noise (90–110 dB SPL, 100–900 ms duration, and 0–1,000 ms interval) with random distribution was generated and introduced via a sound generator and free-field electrostatic speaker (Tucker-Davis Technologies, Alachua, FL, USA). The speaker was located on top of the cage.

### 2.3. MRI Data Acquisition

MRI was performed on mice 24 h after exposure to the white noise. Prior to data acquisition, the mice were anesthetized with isoflurane (1.5% in oxygen) in a chamber and placed in the magnet in the prone position. During data acquisition, anesthesia was maintained (1.0–1.5% isoflurane in oxygen), and respiration and body temperature of the animals were monitored. All MRI data were collected on a 9.4 T MR scanner (Agilent 9.4 T/160AS, Agilent Technologies, Santa Clara, CA, USA) using a single-channel surface coil for both radio-frequency transmission and signal reception. Scout images were acquired using a gradient echo sequence for all three orthogonal directions (repetition time (TR)/echo time (TE) = 29.68/2.99 ms, field-of-view (FOV) = 30 × 30 mm^2^, matrix size = 128 × 128, five slices for each direction (1 mm gap), slice thickness (TH) = 1 mm, receiver bandwidth (BW) = 50 kHz, and 1 signal average). Based on the scout images, T_1_-weighted coronal brain images were acquired using a fast spin-echo sequence (TR/TE = 550/10.7 ms, echo train length (ETL) = 4, FOV = 17 × 17 mm^2^, matrix size = 192 × 192, number of slices = 20 (−0.5 mm gap), TH = 1 mm, receiver BW = 100 kHz, and 16 signal averages). Additional T_1_-weighted brain images were also acquired using a fast spin-echo sequence for the sagittal and horizontal directions (TR/TE = 550/10.7 ms, ETL = 4, FOV = 17 × 17 mm^2^, matrix size = 192 × 192, number of slices = 10 (sagittal; no gap) or 8 (horizontal; no gap), TH = 1 mm, receiver BW = 100 kHz, and 16 signal averages).

### 2.4. MRI Data Analysis

**Figure 2:**
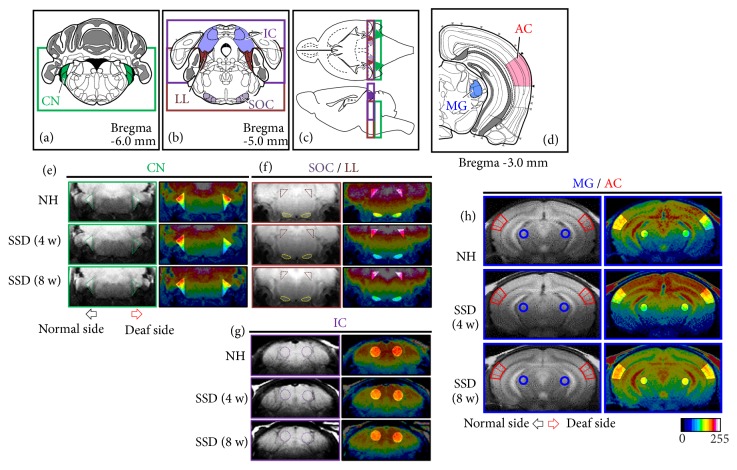
The relative Mn^2+^-enhanced signal intensities (Mn^2+^SI) changes in the CN, SOC, LL, and IC in each group. (a–d) The regions of interest (ROIs) for the CN (a), SOC/LL (b), IC (c), and MG/AC (d). (e–f) T_1_-weighted (left) and color-map (right) images of the slices, including the CN (e), SOC/LL (f), IC (g), and MG/AC (h) in each group, are represented. Image-overlaid triangles (e) and circles (f, g, h) are representative ROIs to quantify signal intensity. Arrows indicate lateral directions of the ipsilateral (red) and contralateral (black) sides. CN, cochlear nucleus; SOC, superior olivary complex; LL, lateral lemniscus; IC, inferior colliculus; MG, medial geniculate body; AC, auditory cortex.

T_1_-weighted MRI data were analyzed quantitatively with ImageJ (v1.47, NIH, http://imagej.nih.gov/ij/). Coronal brain MRIs were used for quantitative analysis of Mn^2+^SI (a.u., arbitrary unit) in defined regions of interest (ROIs; Figure 2) by comparison with a mouse brain atlas (Allen Mouse Brain Atlas, http://mouse.brain-map.org). The ROIs were defined as in a previous study [14]. Before the ROIs were defined, two serial images were rescaled with the RGB scale and were merged by z-projection (with summations by pixel locations) in the stack menu of ImageJ (merged slices included the ROIs: CN, bregma −6.5 to −5.5 mm; SOC, LL, and IC, bregma −5.5 to −4.5 mm; MGB and AC, bregma −3.5 to −2.5 mm). Reconstruction of color-map images is represented in Figure 2 using the 16-color pseudocolor image menu in ImageJ. The measured signal intensities of each ROI were normalized using an internal control (signal intensity of the internal muscle near the brain in each merged slice), which were defined as Mn^2+^SI. The Mn^2+^SI were compared among the groups. The ratios of Mn^2+^SI of the contralateral hearing side to the ipsilateral side were calculated in the CN, SOC, LL, IC, MGB, and AC.

### 2.5. Statistical Analysis

All quantified datasets are presented as the mean ± standard error. Intergroup differences in Mn^2+^SI were analyzed using the ANOVA test and Bonferroni corrections (IBM SPSS, version 21, Armonk, NY, USA). Two-tailed analyses were conducted for interaural differences using the Mann–Whitney* U*-test and the Wilcoxon signed-rank test. Statistical significance was set at* P* < 0.05.

## 3. Results

**Figure 3:**
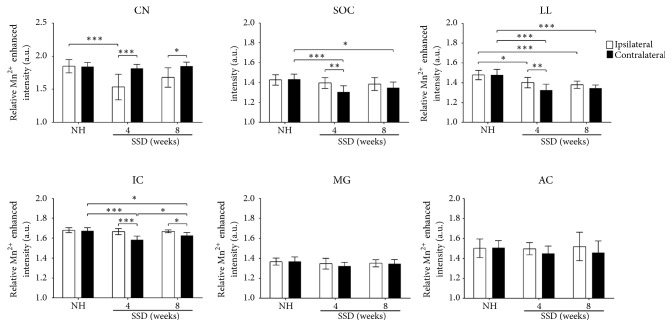
A quantitative analysis of the relative Mn^2+^-enhanced signal intensity (Mn^2+^SI) of each group at the CN, SOC, LL, IC, MG, and AC. In single-sided deafness (SSD) groups, the Mn^2+^SI were decreased in the ipsilateral side of the CN and LL and the contralateral side of the SOC, LL, and IC compared to the control group. The MG and AC did not show any Mn^2+^SI differences. CN, cochlear nucleus; SOC, superior olivary complex; LL, lateral lemniscus; IC, inferior colliculus; MG, medial geniculate body; AC, auditory cortex. *∗P* < 0.05, *∗∗P* < 0.01, and *∗∗∗P* < 0.001, independent and paired* t*-tests.

The ipsilateral side demonstrated decreased Mn^2+^SI at the CN in the SSD-4-week group compared to the control group (*P* < 0.001). On the other hand, the contralateral side of the SSD-4-week group demonstrated significantly lower Mn^2+^SI at the SOC, LL, and IC compared to those of the control group (all* P* < 0.001). The changes in Mn^2+^SI of the ipsilateral CN and contralateral SOC, LL, and IC for the SSD-8-week group were less than those in the SSD-4-week group. However, the contralateral side of the SSD-8-week group still demonstrated significantly lower Mn^2+^SI at the SOC, LL, and IC compared to those of the control group (*P* = 0.011,* P* < 0.001, and* P* = 0.014, respectively). The MG and AC did not show any significant intergroup differences in Mn^2+^SI. It is worth noting that the ipsilateral side of Mn^2+^SI at the LL in the SSD-4-week and SSD-8-week groups also decreased, and the 8-week decrease was bigger than the 4-week change (*P* = 0.002 and* P* < 0.001, respectively) (Figure 3).

**Figure 4:**
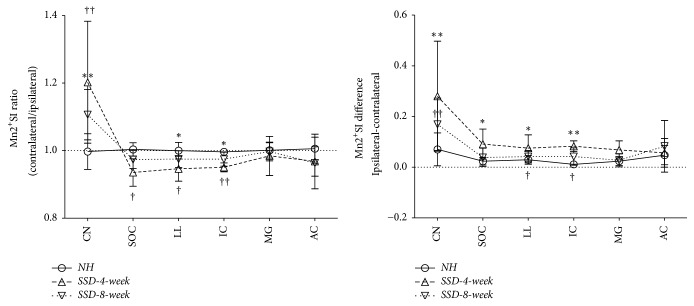
The relative Mn^2+^-enhanced signal intensity (Mn^2+^SI) ratios and absolute differences on the ipsilateral side to contralateral side ears in the CN, SOC, LL, IC, MG, and AC in each group. The interaural Mn^2+^SI ratio was highest in the CN, followed by the SOC, LL, IC, MG, and AC, in the SSD-4-week and SSD-8-week groups. CN, cochlear nucleus; SOC, superior olivary complex; LL, lateral lemniscus; IC, inferior colliculus; MG, medial geniculate body; AC, auditory cortex. *∗*: SSD-4-week group, †: SSD-8-week group, *∗* or †:* P* < 0.05, and *∗∗* or ††:* P* < 0.01, independent and paired* t*-tests.

The contralateral:ipsilateral Mn^2+^SI ratios and absolute differences are presented for each group in Figure 4. The interaural Mn^2+^SI ratio was highest in the CN, followed by the SOC, LL, IC, and MG in the SSD-4-week and SSD-8-week groups. The ipsilateral side of the CN in the SSD-4-week group showed significantly lower Mn^2+^SI than that of the contralateral side (*P* = 0.001). In contrast, the contralateral sides of the SOC, LL, and IC showed significantly lower Mn^2+^SI than those of the ipsilateral sides (*P* = 0.002,* P* = 0.005, and* P* < 0.001, respectively). The MG and AC did not show any significant interaural differences in Mn^2+^SI. Similar interaural Mn^2+^SI differences were detected in the CN, LL, and IC in the SSD-8-week group (*P* = 0.003,* P* = 0.031, and* P* = 0.001, respectively).

## 4. Discussion

### 4.1. Cortical and Subcortical Auditory Neural Activity Changes after SSD

We examined the changes in cortical and subcortical auditory neural activities in accordance with the laterality and duration of SSD using MEMRI. The subcortical neural activities in the CN, SOC, LL, and IC were changed after SSD. Mn^2+^SI decreased significantly on the ipsilateral side of the CN at 4 weeks after SSD, probably due to the deficit of auditory input. In contrast, the higher auditory neural structures, including the SOC, LL, and IC, demonstrated lower Mn^2+^SI on the contralateral side, which can be explained by the decussations of the auditory nervous pathways. Our study strongly supports the decussation pattern found by c-fos expression in the auditory brainstem of mature animals after cochlear ablation [13]. The aural dominance was diluted at higher levels of the auditory nervous system from the CN to the IC. No definite Mn^2+^SI difference was detected in the AC among the groups. In addition, these differences in Mn^2+^SI were alleviated in the SSD-8-week group compared to the SSD-4-week group. Similar to our results, a prior study on unilateral conductive hearing loss in mice demonstrated a significant difference in Mn^2+^SI between the ipsilateral and contralateral side in the IC [14]. However, this previous study performed MEMRI 1 h after unilateral conductive hearing loss, so there was no consideration of the auditory neural plastic changes. Another MEMRI study reported that the Mn^2+^SI in the IC increased proportionally to the amplitude of the sound stimuli [19]. This is the first study that investigated neural activity changes during adaptation after SSD using MEMRI, especially in the subcortical auditory neural system.

### 4.2. Dilution of Aural Dominance in Higher Levels of the Auditory Neural System

The interaural and intergroup difference in Mn^2+^SI was greatest in the CN, and it decreased as it proceeded to higher levels of the auditory neural system (CN > SOC > LL > IC). No significant difference in Mn^2+^SI was found in the AC or MG. Consistent with the present results, some studies have reported comparable auditory neural activities in the AC after SSD [7, 22, 23]. No difference in 2-DG uptake is observed in the AC 3 weeks after unilateral cochlear ablation in gerbils [7]. There are four plausible mechanisms of dilution of aural dominance. First, the accumulation of decussation along the central auditory pathway could result in the dilution of interaural differences at higher levels of the auditory nervous system. As a result, decreased amount of Mn^2+^SI at the contralateral LL was less than that at the SOC. However, decussation may affect both sides of the auditory pathways after SSD. As the contralateral auditory nuclei to be reafferented via decussation, the ipsilateral activity of the SOC and LL, which are thought to be connected to normal auditory input, also showed decreased Mn^2+^SI. Interestingly, the amount of decrease was bigger in the LL than the SOC, and we think that this finding is attributed to the accumulation of decussation as well. Second, the loss of functional inhibition in the contralateral IC might result in increased spontaneous activities in the contralateral AC, resulting in comparable summated neural activities on both sides of the AC. The inhibitory synaptic conductance was decreased in the contralateral IC after SSD in a gerbil study [24]. This loss of inhibition in the contralateral IC may be associated with the attenuated interaural differences in the MG and AC in the MEMRI findings. Third, many bottom-up and top-down neural plastic activities may contribute to the dilution of aural dominance in the higher auditory nervous system. Because integrations of temporal and spectral information of sound and binaural summation are processed in the higher auditory neural system, the differences in Mn^2+^SI might be diluted in the MG and AC. Cortical reorganization, which accompanies the increase in excitatory receptor (AMPA) and the decrease in inhibitory receptor (GABA_A_), leads to the homeostatic neural activity in AC following a month of SSD in mice [23]. The aberrant functional coupling of AC corticofugal neurons with other brain areas, besides the auditory brainstem, is reportedly increased for two weeks following noise-induced hearing loss in mice, according to calcium imaging [25]. Even in the case of deprivation of bilateral auditory input, AC metabolism becomes normalized as the duration of deafness increases [26]. Fourth, because the MEMRI image was acquired after 24 hours of noise exposure, the steady state with saturated Mn^2+^ accumulation might have impeded the differentiation of neural activity changes in high-order auditory brain regions in this study. Other modalities could compensate for the current limitations of MEMRI for auditory cortical activities.

### 4.3. Recovery of Attenuated Subcortical Auditory Neural Activity after SSD

The recovery of the decreased Mn^2+^SI on the ipsilateral side of the CN and contralateral side of the SOC, LL, and IC in the SSD-8-week group compared to the SSD-4-week group suggests that neural activities resumed in the deafferented auditory neural pathways. This finding accords with that of our previous study, which showed recovery of decreased cortical activities during 4–8 weeks after SSD [5]. Continuous auditory stimulation from the normal ear may influence the neural activity of deafferented nuclei along the brainstem because of the binaural interaction components of the subcortical auditory nervous system [27]. The reorganization and spontaneous neural activity of the auditory nervous system may contribute to these neural plastic changes. Spontaneous neural activity increases in the contralateral side of the IC following SSD 2 weeks after acoustic trauma [28]. In addition, the attenuation of contralateral dominance might contribute to the ipsilateral stimulation of the IC from the intact ear. An electrophysiological recording study demonstrated that approximately 2-3 months of SSD resulted in the ipsilateral-dominant activation in the IC [29]. These subcortical auditory neural plastic changes could contribute to the reorganization of the auditory neural system after hearing deprivation at subcortical and cortical levels.

### 4.4. Clinical Implications and Limitations

The reorganized auditory processing circuits may be attributed to the auditory perceptual changes of hyperacusis or tinnitus [30, 31]. In addition, these auditory neural plastic changes could influence the outcome of CI. To unravel the reversibility of brain plastic changes in SSD patients after CI, future study of CI in SSD animals is warranted. Moreover, because different neural plastic changes between prelingual and postlingual SSD patients are predicted, the subcortical neural plastic changes should be explored in young SSD animals.

There were some limitations to this study. The tonotopic mapping of the IC area using MEMRI was suggested in a previous study. However, we could not classify the IC area. Although 24 hours of noise exposure maximized the MEMRI signal:noise ratio, it is possible that MnCl_2_ uptake was saturated and could not discriminate the decreased activities in the AC and MG.

## 5. Conclusion

SSD induced subcortical neural plastic changes in adult mice. Because of the decussation of the auditory neural pathways, neural activities were decreased significantly on the ipsilateral side of the CN and decreased significantly in the contralateral side of the SOC, LL, and IC after SSD. The aural dominance was increasingly diluted at higher levels of the auditory nervous system. This subcortical neural attenuation was partially recovered as the duration of SSD increased. Subcortical auditory neural changes after SSD may contribute to the change in tinnitus severity and the outcomes of cochlear implantation in SSD patients.

## Figures and Tables

**Table 1:** The hearing thresholds of each group (mean ± standard deviation, unit: dB SPL).

	Contralateral (right)	Ipsilateral (left)
Normal hearing (n = 9)	30.00 ± 5.35	29.38 ± 3.20

SSD for 4 weeks (n = 11)	31.82 ± 2.52	81.36 ± 10.02

SSD for 8 weeks (n = 11)	30.71 ± 7.32	80.00 ± 10.00

## Data Availability

The data used to support the findings of this study are available from the corresponding author upon request.

## References

[1] Finke M., Strauss-Schier A., Kludt E., Büchner A., Illg A. (2017). Speech intelligibility and subjective benefit in single-sided deaf adults after cochlear implantation. *Hearing Research*.

[2] Wesarg T., Richter N., Hessel H. (2015). Binaural integration of periodically alternating speech following cochlear implantation in subjects with profound sensorineural unilateral hearing loss. *Audiology and Neurotology*.

[3] Mertens G., De Bodt M., Van de Heyning P. (2016). Cochlear implantation as a long-term treatment for ipsilateral incapacitating tinnitus in subjects with unilateral hearing loss up to 10 years. *Hearing Research*.

[4] Arndt S., Prosse S., Laszig R., Wesarg T., Aschendorff A., Hassepass F. (2015). Cochlear implantation in children with single-sided deafness: Does aetiology and duration of deafness matter?. *Audiology and Neurotology*.

[5] Lee M. Y., Kim D. H., Park S. (2017). Disappearance of contralateral dominant neural activity of auditory cortex after single-sided deafness in adult rats. *Neuroscience Letters*.

[6] Yang M., Chen H., Liu B. (2014). Brain structural and functional alterations in patients with unilateral hearing loss. *Hearing Research*.

[7] Hutson K., Durham D., Imig T., Tucci D. (2008). Consequences of unilateral hearing loss: Cortical adjustment to unilateral deprivation. *Hearing Research*.

[8] Hutson K. A., Durham D., Tucci D. L. (2007). Consequences of unilateral hearing loss: Time dependent regulation of protein synthesis in auditory brainstem nuclei. *Hearing Research*.

[9] Tae W. S., Yakunina N., Kim T. S., Kim S. S., Nam E.-C. (2014). Activation of auditory white matter tracts as revealed by functional magnetic resonance imaging. *Neuroradiology*.

[10] Coomes Peterson D., Schofield B. R. (2007). Projections from auditory cortex contact ascending pathways that originate in the superior olive and inferior colliculus. *Hearing Research*.

[11] Popescu M. V., Polley D. B. (2010). Monaural Deprivation Disrupts Development of Binaural Selectivity in Auditory Midbrain and Cortex. *Neuron*.

[12] Tillein J., Hubka P., Kral A. (2016). Monaural Congenital Deafness Affects Aural Dominance and Degrades Binaural Processing. *Cerebral Cortex*.

[13] Luo L., Ryan A. F., Saint Marie R. L. (1999). Cochlear ablation alters acoustically induced c-fos mRNA expression in the adult rat auditory brainstem. *Journal of Comparative Neurology*.

[14] Yu X., Wadghiri Y. Z., Sanes D. H., Turnbull D. H. (2005). In vivo auditory brain mapping in mice with Mn-enhanced MRI. *Nature Neuroscience*.

[15] Yu X., Sanes D. H., Aristizabal O., Wadghiri Y. Z., Turnbull D. H. (2007). Large-scale reorganization of the tonotopic map in mouse auditory midbrain revealed by MRI. *Proceedings of the National Acadamy of Sciences of the United States of America*.

[16] Duong T. Q., Silva A. C., Lee S.-P., Kim S.-G. (2000). Functional MRI of calcium-dependent synaptic activity: Cross correlation with CBF and BOLD measurements. *Magnetic Resonance in Medicine*.

[17] Mrsic-Flogel T., Hübener M., Bonhoeffer T. (2003). Brain Mapping: New Wave Optical Imaging. *Current Biology*.

[18] Kuo Y.-T., Herlihy A. H., So P.-W., Bell J. D. (2006). Manganese-enhanced magnetic resonance imaging (MEMRI) without compromise of the blood-brain barrier detects hypothalamic neuronal activity in vivo. *NMR in Biomedicine*.

[19] Yu X., Zou J., Babb J. S., Johnson G., Sanes D. H., Turnbull D. H. (2008). Statistical mapping of sound-evoked activity in the mouse auditory midbrain using Mn-enhanced MRI. *NeuroImage*.

[20] Brozoski T. J., Wisner K. W., Odintsov B., Bauer C. A. (2013). Local NMDA receptor blockade attenuates chronic tinnitus and associated brain activity in an animal model. *PLoS ONE*.

[21] Lee H. J., Yoo S.-J., Lee S. (2012). Functional activity mapping of rat auditory pathway after intratympanic manganese administration. *NeuroImage*.

[22] Hanss J., Veuillet E., Adjout K., Besle J., Collet L., Thai-Van H. (2009). The effect of long-term unilateral deafness on the activation pattern in the auditory cortices of French-native speakers: Influence of deafness side. *BMC Neuroscience*.

[23] Balaram P., Hackett T. A., Polley D. B. (2018). Synergistic Transcriptional Changes in AMPA and GABAA Receptor Genes Support Compensatory Plasticity Following Unilateral Hearing Loss. *Neuroscience*.

[24] Vale C., Juíz J. M., Moore D. R., Sanes D. H. (2004). Unilateral cochlear ablation produces greater loss of inhibition in the contralateral inferior colliculus. *European Journal of Neuroscience*.

[25] Asokan M. M., Williamson R. S., Hancock K. E., Polley D. B. (2018). Sensory overamplification in layer 5 auditory corticofugal projection neurons following cochlear nerve synaptic damage. *Nature Communications*.

[26] Ahn S. H., Oh S. H., Lee J. S. (2004). Changes of 2-deoxyglucose uptake in the rat auditory pathway after bilateral ablation of the cochlea. *Hearing Research*.

[27] Laumen G., Ferber A. T., Klump G. M., Tollin D. J. (2016). The Physiological Basis and Clinical Use of the Binaural Interaction Component of the Auditory Brainstem Response. *Ear and Hearing*.

[28] Vogler D. P., Robertson D., Mulders W. H. A. M. (2014). Hyperactivity following unilateral hearing loss in characterized cells in the inferior colliculus. *Neuroscience*.

[29] McAlpine D., Martin R. L., Mossop J. E., Moore D. R. (1997). Response properties of neurons in the inferior colliculus of the monaurally deafened ferret to acoustic stimulation of the intact ear. *Journal of Neurophysiology*.

[30] Yang S., Weiner B. D., Zhang L. S., Cho S.-J., Bao S. (2011). Homeostatic plasticity drives tinnitus perception in an animal model. *Proceedings of the National Acadamy of Sciences of the United States of America*.

[31] Auerbach B. D., Rodrigues P. V., Salvi R. J. (2014). Central gain control in tinnitus and hyperacusis. *Frontiers in Neurology*.

